# Sustaining named GP continuity requires organisational change: a comparative implementation analysis in Finland

**DOI:** 10.3399/BJGP.2026.0036

**Published:** 2026-09-08

**Authors:** Waltteri Tuompo, Janne Mäkelä, Markku Timonen, Juha Auvinen

**Affiliations:** 1 Faculty of Medicine, Research Unit of Population Health, University of Oulu, Oulu, Finland; 2 Wellbeing Services County of North Ostrobothnia, Health Centre of Pudasjärvi, Oulu, Finland; 3 Wellbeing Services County of North Ostrobothnia, Health Centre of Tuira, Oulu, Finland; 4 Wellbeing Services County of North Ostrobothnia, Oulu, Finland

**Keywords:** continuity of patient care, primary health care, general practice, doctor–patient relations, workforce, Finland

## Abstract

**Background:**

Maintaining doctor–patient relationship continuity remains a challenge in public primary care in Finland and internationally owing to GP workforce pressures and organisational constraints.

**Aim:**

To evaluate implementation of a named GP model and explain differences in implementation outcomes between two Finnish primary care units.

**Design and setting:**

A quasi-experimental before–after study was conducted in two Finnish primary care units with contrasting organisational settings. Patients were assigned a named GP responsible for non-urgent care. Electronic health record data covering physician consultations over 48 months were analysed.

**Method:**

Continuity was measured using the Usual Provider of Care (UPC) Index, the St Leonard’s Index of Continuity of Care (SLICC), and the Own Patient Ratio (OPR). Implementation outcomes were interpreted using the RE-AIM framework. A segmented interrupted time series model was applied to OPR and SLICC, and UPC analysed using a pre–post design.

**Results:**

Named GP continuity was higher in Tuira than in Pudasjärvi across all measures. In Tuira, adoption (OPR 0.88) was high and stable with lower named GP-level variation, while reach (SLICC 0.31) and effectiveness (UPC 0.35) improved but remained modest. Although continuity improved in Pudasjärvi, adoption remained weaker (OPR 0.40), reach lower (SLICC 0.16), and maintenance less stable. Continuity of care with physicians other than the named GP increased more in Pudasjärvi.

**Conclusion:**

The named GP model was functionally adopted in Tuira but remained largely nominal in Pudasjärvi. Health systems implementing named GP models should prioritise sustained GP presence and organisational alignment to achieve continuity.

## How this fits in

Continuity of care is associated with better clinical outcomes and lower costs but sustaining it has become increasingly difficult under workforce and organisational pressures. Previous studies suggest that administrative assignment of named GPs alone does not necessarily improve continuity. This study shows that the same named GP reform can succeed in one setting but only partially in another. It demonstrates that interpreting routine continuity measures through an implementation lens can reveal underlying mechanisms, highlighting the importance of stable GP presence, leadership, and process management.

## Introduction

A sustained and trustworthy doctor–patient relationship is a cornerstone of primary health care (PHC). However, GP list systems worldwide have been challenged by rising demand and workforce shortages.^1–3^ As a result, the key question is not whether doctor–patient relationship continuity can be achieved in principle, but how it can be sustained under current constraints and implemented in real-world practice.

Finland had a named GP model in the 1990s that supported continuity, but under-resourcing led to unsustainable workloads, GP attrition, and its collapse by the early 2000s.^4^ Since then, Finnish PHC has shifted towards larger, multidisciplinary health centres — some serving >100 000 residents — mirroring trends in the UK, where an emphasis on timely access and increasingly episodic care has been perceived as compromising continuity.^5,6^ In this respect, Finland may serve as an early example of the long-term consequences of sustained pressure on doctor–patient relationship continuity, offering lessons for systems facing similar challenges.

Over the past 25 years, both continuity and access to GP services have declined in Finland,^7^ while PHC resources have remained largely unchanged and secondary care resources have more than doubled.^8^ Finnish PHC nevertheless remains relatively expensive in international comparisons: in 2024, outpatient expenditure was €329 (approximately 281 GBP) per capita,^9^ about 60% higher than Denmark’s €207 (approximately 178 GBP) for general practice,^10^ a system known for good access and continuity. Finland also ranks among European Union countries with the highest unmet medical need, particularly among low-income residents.^11^

Only one-third of Finnish PHC physicians are GP specialists; the remainder are GP registrars or locums.^12^ Patients cannot book GP appointments directly; instead, nurses act as gatekeepers and refer to physicians when deemed necessary, creating barriers amid GP shortages. Many patients are managed by nurses with indirect physician input (paper consultations) or directed to same-day urgent visits with whichever physician is on duty. Even when non-urgent appointments are available, they are often allocated to any physician with open slots rather than the patient’s regular physician.

The benefits of doctor–patient relationship continuity are well established: it has been consistently associated with lower demand,^5,13^ lower costs,^14^ better outcomes,^15–19^ and reduced mortality.^15,18,20^ In Finland, expanding contacts with non-GP personnel has not improved access to GP services and may have increased administrative workload, reflected in the halving of physician consultation volumes over two decades.^7^ Similar challenges have been reported in UK general practice, where expanding non-GP roles has not consistently translated into improved access.^5^

Given this context, it remains unclear not simply whether named GP models improve continuity, but whether continuity reforms can be implemented and sustained under current constraints in GP availability and access pressures, and whether they perform similarly across PHC settings. This study therefore evaluated the implementation of a named GP model in two Finnish PHC centres and examined how implementation differences affected doctor–patient relationship continuity.

## Method

### Study design and population

This quasi-experimental before–after study compared implementation of a named GP model in two Finnish public health centres, Pudasjärvi and Tuira, in the Wellbeing Services County of North Ostrobothnia. Pudasjärvi is a rural municipality of about 7500 residents (1.28/km²), and Tuira an urban district of Oulu with about 65 000 residents (>1028/km²).

At implementation, all residents were listed to named GPs, comprising the study population (Figure 1). Data included all physician consultations over a 48-month period (24 months before and after implementation), including date, consultation type, and physician and patient identification (ID). Data were extracted anonymously from the regional data pool.

**Figure 1.**
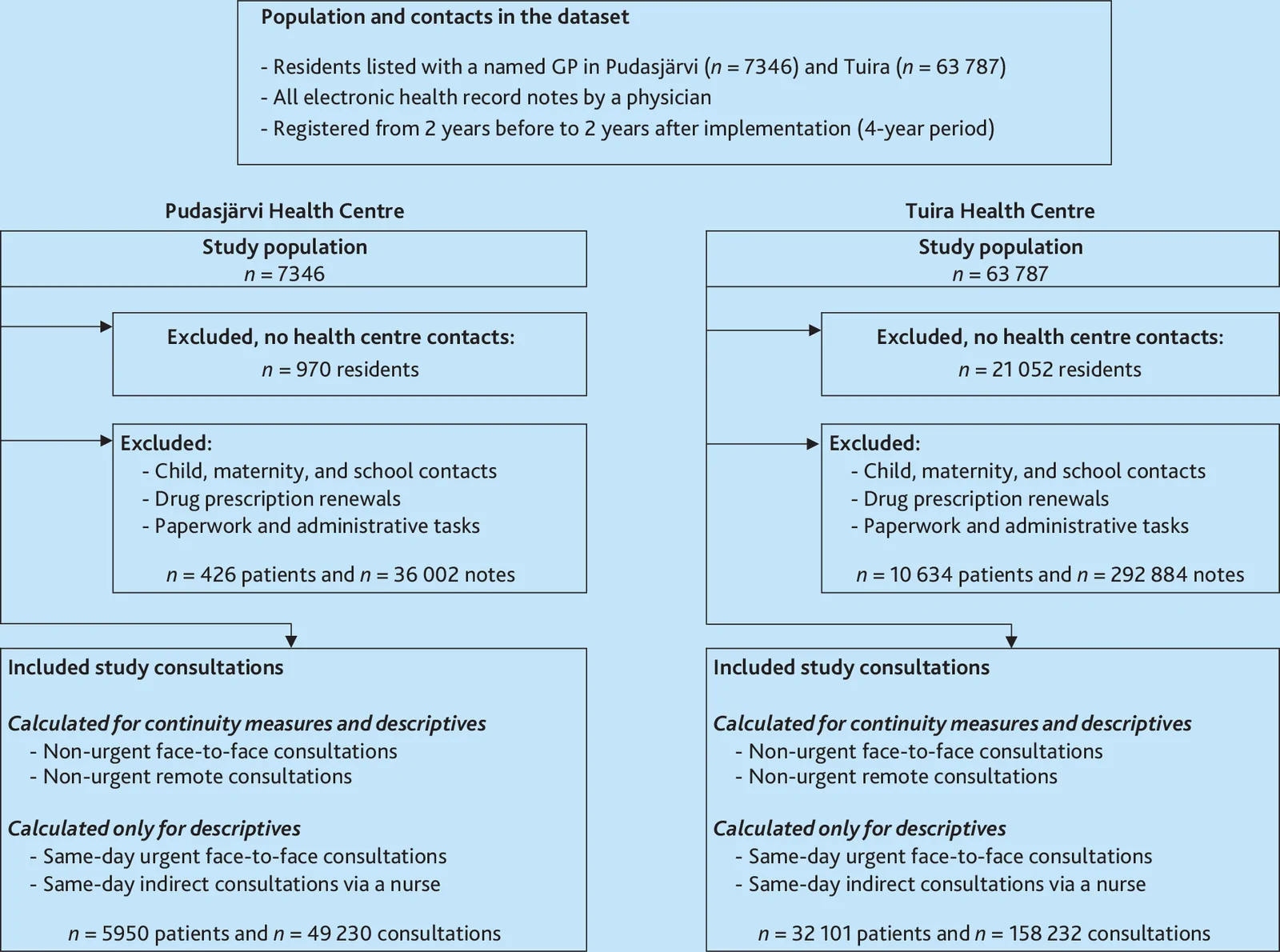
Flow of patient and consultation selection for analysis in the two study units.

### Description of the implemented model

In Tuira, residents and physicians were previously organised into four teams responsible for non-urgent care, while urgent consultations were centralised. The named GP model was introduced in team 2 in October 2022 and expanded to teams 1, 3, and 4 by February 2023. In Pudasjärvi, the model was implemented in April 2023.

Non-urgent consultations were previously typically assigned to the next available physician. Under the named GP model, patients were allocated to a specific GP or GP registrar, prioritising existing care relationships when possible and otherwise using alphabetical assignment. The named GP was recorded in the electronic health records and displayed during appointment booking to guide routing. Most GPs worked part-time, and list sizes were adjusted to estimated working time, ranging from 1200 to 2900 residents per GP in Tuira and 550 to 1100 in Pudasjärvi.

The named GP model operated alongside routine health centre practice, in which physicians’ duties varied daily. Local chief physicians led the health centres, overseeing workforce allocation and implementation of the named GP model. Named GPs provided non-urgent consultations (face-to-face and remote) and same-day consultations (urgent face-to-face and paper-based) in addition to other responsibilities outside the model, including maternity, child health and school clinics, ward work, residential care homes, supervision, training, and mandatory hospital rotations for GP registrars. As a result, named GPs were not continuously available for non-urgent care, and absences were intended to be covered by locums.

Continuity analyses focused on non-urgent consultations, excluding urgent consultations and other GP responsibilities, as the named GP model applied only to this care stream. Further details on the Finnish primary care context and organisational constraints are provided in the Supplementary Information S1–S5.

### Framework, rationale, and analytical structure

The evaluation was structured using the RE-AIM framework to organise and interpret outcomes related to reach, effectiveness, adoption, implementation, and maintenance of the named GP model, using an implementation lens.^21^ RE-AIM was used to conceptualise outcomes and to support interpretation of how and why continuity effects diverged between settings.

Guided by this framework, three quantitative continuity indicators were used: the Usual Provider of Care (UPC) Index, the St Leonard’s Index of Continuity of Care (SLICC), and the Own Patient Ratio (OPR).^22^ These indicators captured complementary aspects of continuity at different analytic levels: UPC measured longitudinal continuity at the patient level, SLICC the monthly proportion of consultations with the named GP at the list-population level, and OPR the proportion delivered by a GP to their own listed patients at the GP level. Measures were based on GP consultations only. Box 1 outlines the operationalisation of the RE-AIM dimensions; detailed definitions are provided in Supplementary Table S1.

**Box 1.** Mapping of continuity of care indicators to the RE-AIM framework

RE-AIM dimensions	Definition	Used indicators	Analytical approach	Interpretation in the context of the named GP model
**Reach**	Proportion of the target population who received the intervention or were covered by it	SLICC	Segmented ITS *- Basic model* *- Adjusted for named GP activity*	Describes the population-level reach of the model, representing the share of consultations that occurred with patients’ own named GPs. In the adjusted model, the activity moderator captures whether GP availability influenced the model’s reach.
**Effectiveness**	Impact of the intervention on key outcomes among those exposed	UPC	Pre–post comparison *- Paired samples*	Patient-level continuity indicator describing the model’s effectiveness in maintaining ongoing care relationships with the named GP among regular service users (≥2 consultations during 24-month follow-up).
**Adoption**	Proportion and representativeness of settings or providers who adopted the intervention	OPR	Segmented ITS *- Basic model* *- Adjusted for named GP activity*	Represents the proportion of each GP’s consultations conducted with their own listed patients, describing how widely the model was adopted among named GPs. In the adjusted model, the activity moderator captures whether GP availability influenced the model’s adoption.
**Implementation**	Fidelity and consistency across settings	Gini (SLICC and OPR)	Post-implementation variation analysis *- From named GPs monthly SLICC and OPR*	Assesses how consistently the named GP model was delivered among GPs and their patient lists within each unit. Variation metrics (from monthly SLICC and OPR values) quantify post-implementation disparities across providers and patient lists, indicating the overall level of implementation consistency.
**Maintenance**	Describes how the intervention is sustained over time	SLICC and OPR	Segmented ITS *- Basic model* *- Adjusted for named GP activity*	Evaluates whether the adoption and continuity achieved by the model were sustained after implementation. The post-intervention trend indicates the model’s long-term stability, and the activity adjustment captures whether GP availability influenced its maintenance over time.

Gini = measure of variation in SLICC and OPR across named GPs during the post-implementation period (0 = equal, 1 = highly unequal). ITS = Interrupted Time Series. OPR = Own Patient Ratio. SLICC = St Leonard’s Index of Continuity of Care. UPC = Usual Provider of Care Index.

### Statistical analysis

Segmented interrupted time series (ITS) models were fitted to 48 monthly GP-level SLICC and OPR observations (24 pre- and 24 post-intervention) using weighted least squares with consultation counts as weights, including terms for immediate post-intervention change and post-intervention trend. Sensitivity analyses showed robustness to seasonality and lag specification. Trajectories were illustrated using fitted values with heteroskedasticity- and autocorrelation-consistent (HAC) standard errors. Post-implementation variation for SLICC and OPR was assessed using weighted Gini coefficients.

In adjusted ITS models, the interaction term captured the moderating effect of GP availability. Availability was defined as the proportion of months with at least one non-urgent consultation, distinguishing regularly present GPs from those with prolonged absences (for example, GP registrars undertaking hospital rotations as a part of specialisation).

For UPC, pre–post comparisons used paired samples of patients with ≥2 consultations per period, calculated for both the named GP and the most frequently seen physician, while 95% confidence intervals (CI) were estimated using non-parametric bootstrapping.

Descriptive statistics summarised the population size, age, and sex distribution, and numbers of users and consultations. Table 1 also reports consultation-mix indicators. Analyses were conducted in Python (version 3.12).

**Table 1.** Characteristics of users, consultations, and GP workload before and after implementation

Characteristic	Tuira pre	Tuira post	Pudasjärvi pre	Pudasjärvi post
**Population or users**				
Total population (listed patients), *n*	63 787	63 787	7346	7346
Named GPs, *n*	31	31	9	9
Health-centre users with ≥1 consultations, *n* (%)	22 526 (35.3)	24 950 (39.1)	4950 (67.4)	4863 (66.2)
Age of users, years, mean (95% CI)	41.4 (41.1 to 41.8)	41.0 (40.6 to 41.3)	50.9 (50.1 to 51.7)	50.7 (49.9 to 51.6)
Age of users, years, median (IQR)	41.1 (22.6–66.2)	39.6 (21.8–65.8)	59.1 (31.1–72.5)	59.7 (30.7–72.4)
Male users, %	41.9	41.3	49.6	48.9
**Non-urgent consultations**				
Consultations, *n*	31 100	35 833	9617	8227
Patients with ≥1 non-urgent consultations, *n*	14 268	16 336	3255	2909
Patients with ≥2 consultations (independent), *n*	7225	8520	2073	1729
Paired patients with ≥2 consultations, *n*	3726	3726	1061	1061
Physician consultations per patient, mean (95% CI)	2.18 (2.15 to 2.21)	2.19 (2.17 to 2.22)	2.95 (2.86 to 3.04)	2.83 (2.72 to 2.93)
Age, years, mean (95% CI)	47.8 (47.3 to 48.2)	46.6 (46.2 to 47.0)	57.8 (56.9 to 58.7)	57.6 (56.7 to 58.6)
Age, years, median (IQR)	51.7 (29.0–70.3)	49.9 (27.5–69.2)	66.0 (45.9–75.6)	65.7 (47.1–75.0)
**Same-day consultations**				
Consultations, *n*	43 153	48 146	13 857	17 529
Patients with ≥1 same-day urgent consultations, *n*	17 517	19 690	4 235	4 479
Consultations per patient, mean (95% CI)	2.46 (2.42 to 2.50)	2.45 (2.41 to 2.48)	3.27 (3.17 to 3.37)	3.91 (3.79 to 4.04)
Age, years, mean (95% CI)	40.5 (40.1 to 40.9)	40.6 (40.2 to 41.0)	50.1 (49.3 to 51.0)	50.7 (49.9 to 51.5)
Age, years, median (IQR)	38.7 (21.7–66.0)	39.0 (21.2–66.0)	58.0 (29.6–72.3)	59.7 (30.2–72.7)
**Workload or overall mix**				
Share of consultations that were non-urgent, %	41.9	42.7	41.0	31.9
Share of non-urgent consultations performed by named GPs, %	37.7	36.4	42.2	40.0
Non-urgent / named GP / mo., mean (95% CI)	15.8 (12.7 to 18.6)	17.5 (14.2 to 20.7)	18.8 (15.7 to 22.3)	15.2 (8.5 to 21.6)
Non-urgent / named GP / active mo., mean (95% CI)	23.2 (20.7 to 25.8)	22.1 (19.5 to 24.7)	25.5 (21.0 to 30.5)	23.6 (18.9 to 27.6)
Share of same-day performed by named GPs, %	45.0	51.1	55.9%	37.4
Same-day / named GP / mo., mean (95% CI)	26.1 (19.6 to 31.9)	33.1 (24.3 to 40.6)	35.9 (26.5 to 44.6)	30.4 (18.2 to 42.5)
Same-day / active GP / active mo., mean (95% CI)	36.6 (31.3 to 41.7)	38.1 (31.0 to 44.3)	48.5 (36.3 to 59.5)	40.5 (28.2 to 53.7)

Active GP/active mo. = months in which the named GP performed ≥1 consultation in the given category. IQR = interquartile range. mo. = mean per calendar month across the 24-month period. Performed by named GPs = proportion of all consultations conducted by any GP who was assigned as a named GP, regardless of whether the patient belonged to their personal list. Post = 24-month period after implementation. Pre = 24-month period before the implementation of the continuity model.

## Results

### Descriptive characteristics

The Tuira population was younger than Pudasjärvi’s (mean age 41 years versus 51 years). Service use was higher in Pudasjärvi, where about two-thirds of residents used GP services compared with about one-third in Tuira. After implementation, named GPs accounted for a slightly larger share of non-urgent consultations in Pudasjärvi (40%) than in Tuira (36%). Monthly consultations per active named GP were higher for same-day than non-urgent care (41 versus 24 in Pudasjärvi; 38 versus 22 in Tuira), indicating that most outpatient contacts occurred outside the model (Table 1).

### ITS model performance

Model diagnostics indicated adequate fit for SLICC (*R*² = 0.51–0.53) and OPR (*R*² = 0.78–0.84) ITS models. Durbin–Watson tests indicated no substantial autocorrelation, except in the Tuira OPR model (0.58), which was addressed using HAC (see Supplementary Tables S1–S4).

### RE-AIM dimensions

#### Reach

SLICC increased in both units, with larger gains in Tuira than in Pudasjärvi. In Tuira, the post-intervention level change was +0.10 (95% CI = 0.05 to 0.16), reaching a fitted value of 0.31. In Pudasjärvi, the corresponding level change was +0.04 (95% CI = 0.02 to 0.08), reaching 0.16. Availability-adjusted effects were +0.07 (95% CI = 0.03 to 0.10) in Tuira and +0.03 (95% CI = 0.00 to 0.05) in Pudasjärvi, with larger effects among GPs with more stable availability (+0.05 in Tuira; +0.02 in Pudasjärvi) (Figure 2, Table 2, and Supplementary Table S2).

**Figure 2.**
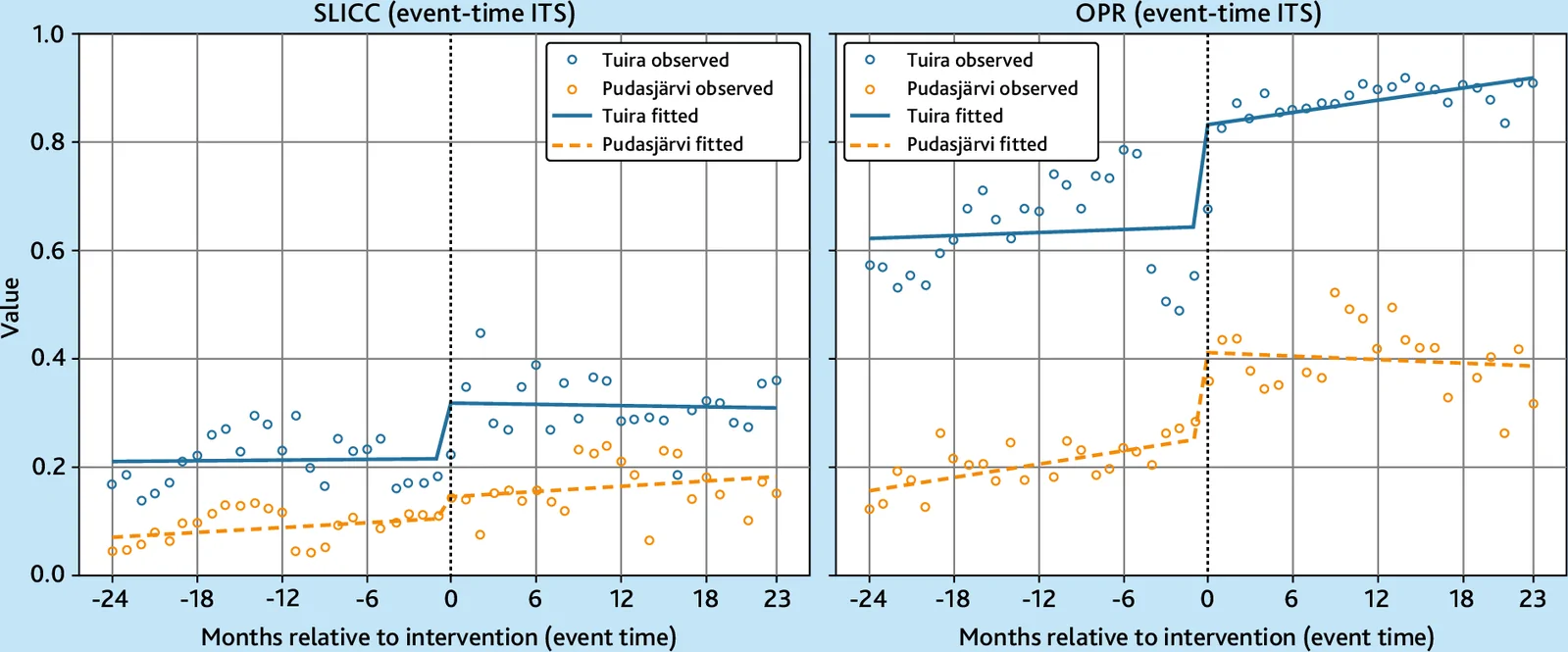
Fitted interrupted time series (ITS) trajectories for SLICC and OPR in Tuira and Pudasjärvi. OPR = Own Patient Ratio. SLICC = St Leonard’s Index of Continuity of Care.

**Table 2.** Summary of results across RE-AIM dimensions^a^

**RE-AIM dimension**	**Indicator**	**Analytical approach**	**Tuira, main results**	**Pudasjärvi, main results**	**Interpretation summary**
Reach	SLICC	Segmented ITS	SLICC:0.21 (95% CI = 0.19 to 0.23) → 0.31 (95% CI = 0.29 to 0.33)	SLICC:0.09 (95% CI = 0.08 to 0.10) → 0.16 (95% CI = 0.14 to 0.18)	Coverage with patients’ own named GPs increased in both units after implementation, with higher absolute levels in Tuira. Adjustment for GP availability indicated that stable provider presence partly explained the improvement.
			β_post (basic):0.10 (95% CI = 0.05 to 0.16)	β_post (basic):0.04 (95% CI = 0.02 to 0.08)	
			β_post (adjusted):0.07 (95% CI = 0.03 to 0.10)	β_post (adjusted):0.03 (95% CI = 0.00 to 0.05)	
			β_post×avail (adjusted):0.05 (95% CI = 0.03 to 0.08)	β_post×avail (adjusted):0.02 (95% CI = 0.00 to 0.04)	
Effectiveness	UPC	Pre–post comparison	Named GP:0.21 (95% CI = 0.20 to 0.23) →0.35 (95% CI = 0.34 to 0.36)	Named GP:0.08 (95% CI = 0.07 to 0.09) →0.16 (95% CI = 0.14 to 0.18)	Patient-level continuity improved in both units. In Tuira, a larger share of the improvement was attributable to consultations with patients’ own named GPs. In Pudasjärvi, overall continuity increased, but continuity with named GPs remained low, suggesting a more heterogeneous or partial effect of the model.
			Most frequent physician:0.58 (95% CI = 0.57 to 0.59) →0.61 (95% CI = 0.60 to 0.62)	Most frequent physician:0.46 (95% CI = 0.44 to 0.48) →0.64 (95% CI = 0.62 to 0.66)	
Adoption	OPR	Segmented ITS	OPR:0.64 (95% CI = 0.60 to 0.67) →0.87 (95% CI = 0.86 to 0.89)	OPR:0.20 (95% CI = 0.19 to 0.21) →0.40 (95% CI = 0.40 to 0.40)	Adoption was strong and widespread in Tuira, with high absolute OPR levels. In Pudasjärvi, adoption was more modest but showed clear relative gains. Greater effects among consistently available GPs suggest that stable provider presence supported uptake.
			β_post (basic):0.18 (95% CI = 0.05 to 0.31)	β_post (basic):0.16 (95% CI = 0.10 to 0.22)	
			β_post (adjusted):0.12 (95% CI = 0.03 to 0.20)	β_post (adjusted):0.11 (95% CI = 0.07 to 0.15)	
			β_post×avail (adjusted):0.09 (95% CI = 0.03 to 0.15)	β_post×avail (adjusted):0.08 (95% CI = 0.05 to 0.11)	
Implementation	Gini coefficient	Post-implementation variation analysis	OPR Gini:0.07 (95% CI = 0.06 to 0.08)	OPR Gini:0.17 (95% CI = 0.12 to 0.22)	Post-implementation variation was lower in Tuira for provider adoption (OPR), reflecting more consistent engagement across physicians. At the population level (SLICC), Tuira showed broader but more even distribution, whereas Pudasjärvi displayed higher inequality, with continuity concentrated among fewer GPs.
			SLICC Gini:0.42 (95% CI = 0.39 to 0.45)	SLICC Gini:0.49 (95% CI = 0.45 to 0.52)	
Maintenance	SLICC, OPR	Segmented ITS	SLICC β_time_after (basic): –0.000 (95 % CI = –0.006 to 0.004)	SLICC β_time_after (basic):0.000 (95% CI = –0.002 to 0.003)	Post-intervention levels remained stable in Tuira, while in Pudasjärvi the OPR trend showed a slight but statistically significant decline, suggesting a modest loss of continuity over time after implementation.
			OPR β_time_after (basic):0.003 (95% CI = -0.007 to 0.013)	OPR β_time_after (basic):0.003 (95% CI = –0.007 to 0.013)	
			SLICC β_time_after (adjusted): –0.000 (95% CI = –0.05 to 0.04)	SLICC β_time_after (adjusted):0.000 (95% CI = –0.002 to 0.003)	
			OPR β_time_after (adjusted):-0.002 (95% CI = –0.005 to 0.004)	OPR β_time_after (adjusted): –0.005 (95% CI = –0.008 to –0.002)	

^a^Segmented interrupted time series (ITS) and pre–post analyses were conducted to evaluate the named GP model. Reported estimates show coefficients (β) for basic and adjusted models with 95% confidence intervals, and fitted absolute levels where applicable. Adjusted = model including GP availability interaction term. Basic = model without availability adjustment. β_post = immediate level change after implementation. β_post×avail = interaction between post-implementation period and GP availability. β_time_after = change in trend after implementation. Gini = measure of variation across GPs during the post-implementation period (0 = equal, 1 = highly unequal). OPR = Own Patient Ratio. SLICC = St Leonard’s Index of Continuity of Care. UPC = Usual Provider of Care Index.

#### Effectiveness

UPC with the named GP increased in both units, remaining consistently higher in Tuira. In Tuira, mean UPC with named GP increased from 0.21 (95% CI = 0.20 to 0.23) to 0.35 (95% CI = 0.34 to 0.36); in Pudasjärvi, from 0.08 (95% CI = 0.07 to 0.09) to 0.16 (95% CI = 0.14 to 0.18). UPC with the most frequently seen physician increased more in Pudasjärvi than in Tuira (Table 2 and Supplementary Table S5).

#### Adoption

OPR increased in both units, with higher absolute levels in Tuira. The post-intervention level change was +0.18 (95% CI = 0.05 to 0.31), reaching 0.87 in Tuira, and +0.16 (95% CI = 0.10 to 0.22), reaching 0.40 in Pudasjärvi. Availability-adjusted models showed positive effects in Tuira (+0.12, 95% CI = 0.03 to 0.20) and in Pudasjärvi (+0.11, 95% CI = 0.07 to 0.15), with stronger effects where GP presence was more stable (+0.09 in Tuira; +0.08 in Pudasjärvi) (Figure 2, Table 2, and Supplementary Table S3).

#### Implementation

Implementation was more consistent in Tuira than in Pudasjärvi, with lower OPR variation (Gini 0.07, 95% CI = 0.06 to 0.08 versus 0.17, 95% CI = 0.12 to 0.22). SLICC showed higher overall variation but the same direction (Gini 0.42, 95% CI = 0.39 to 0.45 versus 0.49, 95% CI = 0.45 to 0.52) (Table 2).

#### Maintenance

Post-intervention trends did not change significantly in Tuira, indicating sustained effects. In Pudasjärvi, the adjusted model showed a small but statistically significant decline in OPR (–0.005, 95% CI = –0.008 to –0.002), indicating a modest reduction in adoption (Figure 2, Table 2, and Supplementary Table S3).

## Discussion

### Summary

This study evaluated the implementation of a named GP model in two Finnish health centres. Although continuity improved across multiple indices in both units, the magnitude and sustainability of gains differed. Application of the RE-AIM framework^22^ indicated that similar population-level continuity gains reflected different mechanisms: functional implementation in Tuira versus more nominal adoption in Pudasjärvi.

Named GP continuity improved in both settings but remained substantially higher in Tuira. In Pudasjärvi, continuity measured by the most frequently seen physician increased more despite weaker adoption metrics. Because UPC is sensitive to visit frequency and cannot reach zero, when based on the most frequent physician, differences in consultation frequencies and volume may influence absolute levels. The increase may also reflect organisational practices and short-term episodic clustering of 2–3 visits with the same physician, including locum physicians, suggesting that continuity developed outside the named GP structure rather than through sustained GP–patient relationships.

Adoption metrics supported this interpretation. In Tuira, high and stable OPR values indicated that patient allocation and nurse-led appointments booking routing to named GPs functioned largely as intended, suggesting that further gains in other implementation dimensions would depend primarily on increasing named GP capacity. In Pudasjärvi, weaker adoption and declining OPR suggest that observed continuity gains were not primarily driven by strengthened list-based structure.

Higher pre-intervention continuity levels in Tuira indicated that list formation incorporated existing care relationships more deliberately. Nevertheless, even in Tuira — where adoption was considered successful — relatively low consultation frequencies and only moderate reach (proportion of visits conducted by named GPs) suggest that formal list allocation alone may be insufficient.

Measured continuity appears to depend not only on list allocation but also on organisational processes that support access to the named GP, including whether nursing staff are instructed to book appointments with the named GP, as well as ensuring sufficient protected clinical time for the named GP role. However, it remains questionable whether real, effective continuity can be achieved when continuity is limited only to a small number of non-urgent consultations, where usually also waiting times are long. In Finland, this has led to a need for nurses to act as gatekeepers, which further reduces opportunities for the doctor–patient relationship to develop and for achieving the well-documented benefits of continuity of care.

Overall, these findings indicate that structural doctor–patient relationship continuity reforms succeed only when supported by effective implementation, including clear planning, leadership, and aligned processes. Despite improvements, absolute continuity levels remained modest. Therefore, further research is needed to determine whether these changes translate into clinically meaningful outcomes. Our findings offer lessons for health systems facing similar organisational constraints.

### Strengths and limitations

The study used comprehensive electronic health record data covering all physician consultations over 4 years, ensuring full population coverage at both sites. Multiple continuity indicators provided a multidimensional view of implementation. The clearly defined intervention enabled detailed examination under routine conditions and offered deeper local insight than large registry studies, albeit with more limited generalisability. Including two comparable yet contextually distinct health centres allowed examination not only of whether the reform worked, but also how and why implementation differed across settings, enhancing policy relevance.

The study has limitations. It relied solely on quantitative indicators; qualitative factors such as leadership, staff engagement, and GP and patient experience, including patient-reported experience and outcome measures, were not examined. Patient cohorts were defined at implementation, and subsequent list changes could not be tracked but were likely minor. Because only two health centres were studied, conclusions about the influence of unit size must be drawn cautiously. The absence of an external control limits causal inference, although the two units served as natural comparators. Finally, analyses were restricted to non-urgent consultations and therefore reflect continuity within non-urgent care rather than across the full scope of primary care.

### Comparison with existing literature

Previous qualitative studies in Finland have shown that having a named GP is associated with higher continuity,^23^ whereas discontinuing named GP models reduces it.^24^ Recently, team-based models have shown mixed effects on continuity.^25^ To the authors’ knowledge, this is the first Finnish study to examine the implementation of a named GP model, including its uptake and sustainability, and its effects on continuity.

International studies associate continuity with higher GP productivity,^14^ lower healthcare utilisation,^14–17,19^ and reduced mortality.^15,18,19^ However, the continuity levels observed here are not directly comparable with large registry studies covering broader primary care, as this study focused on implementing a named GP model in non-urgent consultations.

In Canada, formal patient enrolment has been associated with higher continuity,^26^ whereas in Sweden and the UK, limiting named GP assignment to older or chronically ill patients did not improve continuity.^27,28^ Recent evaluations therefore emphasise the importance of operationalising continuity through practice-level change, leadership, and measurement systems.^3^ The present findings align with this evidence and extend it by demonstrating that variation in local implementation determines success.

Strengthening continuity of care has become an important policy objective in several countries beyond Finland. For example, in England, recent changes to the GP contract emphasise contractual mechanisms, including the use of risk stratification tools to support continuity,^29^ while in Sweden legislation requires primary care to be organised so that patients can choose a provider and have access to a named doctor.^30^ The present findings suggest that in systems where continuity is organised at the level of care provider organisations, rather than through direct patient enrolment with a personally selected GP, improvements are unlikely to occur automatically. Instead, successful implementation is likely to depend on targeted interventions and the incentives and engagement of local operational leadership.

### Implications for research and practice

This study shows that a named GP model improves continuity only when implemented as genuine organisational change rather than a nominal administrative measure. Weaker adoption in Pudasjärvi highlights that implementation quality, rather than context alone, is decisive. Sustained implementation requires embedding list-based working into routine practice through stable GP presence, protected time, and consistent workforce allocation. Further research should examine the mechanisms underlying successful implementation; for example, through qualitative process evaluation of organisational routines, leadership, and interprofessional collaboration.

For policymakers, existing evidence strongly supports the value of doctor–patient relationship continuity, leaving little justification for not pursuing named GP policies. This study demonstrates that such policies are feasible under current constraints; however, without corresponding operational change in day-to-day practice, they risk creating false reassurance rather than the organisational structures needed to support long-term doctor–patient relationship continuity.

The findings further suggest that the success of continuity reforms is likely to depend on how they are implemented. Depending on system context, policy instruments should incentivise continuity at the level at which care is organised and delivered — whether through organisational leadership, team-level practices, or individual GPs. While the specific mechanisms may vary between healthcare systems, attention to implementation at the appropriate level appears crucial to achieving sustained improvements in doctor–patient relationship continuity.

## Data

Data are not publicly available due to privacy and ethical restrictions; access was limited to the research team under the conditions of the research permission granted by the Wellbeing Services County of North Ostrobothnia.
